# Associations of insulin resistance and inflammatory biomarkers with endometrial cancer survival: The Alberta endometrial cancer cohort study

**DOI:** 10.1002/cam4.4584

**Published:** 2022-02-16

**Authors:** Andria R. Morielli, Renée L. Kokts‐Porietis, Jamie L. Benham, Jessica McNeil, Linda S. Cook, Kerry S. Courneya, Christine M. Friedenreich

**Affiliations:** ^1^ Department of Cancer Epidemiology and Prevention Research, Cancer Care Alberta Alberta Health Services Calgary Alberta Canada; ^2^ Department of Medicine, Cumming School of Medicine University of Calgary Calgary Alberta Canada; ^3^ Department of Community Health Sciences, Cumming School of Medicine University of Calgary Calgary Alberta Canada; ^4^ Department of Kinesiology, Faculty of Health and Human Sciences University of North Carolina Greensboro Greensboro North Carolina USA; ^5^ Department of Epidemiology Colorado School of Public Health Aurora Colorado USA; ^6^ Faculty of Kinesiology, Sport, and Recreation University of Alberta Edmonton Alberta Canada; ^7^ Department of Oncology, Cumming School of Medicine University of Calgary Calgary Alberta Canada

**Keywords:** biomarkers, endometrial cancer, inflammation, insulin resistance, mortality, survival

## Abstract

**Background:**

Metabolic dysfunction and inflammation have been associated with endometrial cancer risk; however, their influence on endometrial cancer survival is less understood.

**Methods:**

A prospective cohort study of 540 endometrial cancer cases diagnosed between 2002 and 2006 in Alberta were followed for survival outcomes to 2019. Baseline blood samples collected either pre‐ or post‐hysterectomy were analyzed for glucose, insulin, adiponectin, leptin, tumor necrosis factor‐α, interleukin‐6, and C‐reactive protein. Covariates were obtained during in‐person interviews and via medical chart abstraction. Cox proportional hazard regression models were used to estimate multivariable‐adjusted hazard ratios (HR) and 95% confidence intervals (95% CI) for the association between each biomarker and disease‐free and overall survival.

**Results:**

Blood samples were collected from 520 of the 540 participants (presurgical *n* = 235; postsurgical *n* = 285). During the median follow‐up of 14.3 years (range 0.4–16.5 years), there were 125 recurrences, progressions, and/or deaths with 106 overall deaths. None of the biomarkers were associated with disease‐free or overall survival in multivariable‐adjusted analyses. In an exploratory stratified analysis, the highest level of presurgical adiponectin, compared to the lowest level, was associated with improved disease‐free (HR = 0.42, 95% CI = 0.20–0.85) and overall (HR = 0.41, 95% CI = 0.18–0.92) survival, whereas no statistically significant associations were noted for postsurgical measures of adiponectin.

**Conclusions:**

Overall, there was no evidence of an association between biomarkers of insulin resistance and inflammation with mortality outcomes in endometrial cancer survivors. Future cohort studies with serial blood samples are needed to understand the impact of changes in insulin resistance and inflammatory markers on endometrial cancer survival.

## Graphical abstract

The Alberta Endometrial Cancer Cohort Study prospectively examined the association between biomarkers of insulin resistance and inflammation with survival outcomes in 540 incident endometrial cancer cases ascertained through the Alberta Cancer Registry between 2002 and 2006. Shortly after their cancer diagnosis, participants provided an 8‐hour fasting blood sample and completed in‐person interviews during which detailed demographic, medical and lifestyle information was collected. Clinical data were abstracted from medical records and vital status was obtained by the Alberta Cancer Registry through data linkage with Vital Statistics Alberta and Statistics Canada to 2019.

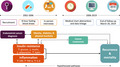

## 1 INTRODUCTION

Endometrial cancer is the sixth most common cancer in women worldwide.^1^ In 2020, approximately 417,367 women were diagnosed with endometrial cancer and 97,370 died from the disease.^1^ Moreover, in the United States, endometrial cancer is one of the few cancers with both increasing incidence (about 1% annually from 2007 to 2016) and mortality rates (about 2% annually from 2008 to 2017).^2^ In Canada, the incidence rate for endometrial cancer has remained relatively stable since 2001 still, the mortality rate has increased by about 2% every year since 1984.^3^

Metabolic dysregulation and chronic inflammation promote carcinogenesis by reducing cancer cell apoptosis and by increasing cancer cell growth, angiogenesis, metastasis, and resistance to cancer treatments.^4^, ^5^ Established risk factors for endometrial cancer include obesity, type 2 diabetes, smoking, and physical inactivity.^6^, ^7^ Moreover, there is evidence, including data from our population‐based case–control study,^8^, ^9^ to support the mechanistic role of insulin resistance and inflammation in the development of endometrial cancer.^10^, ^11^, ^12^, ^13^, ^14^, ^15^, ^16^ Insulin resistance has been positively associated with endometrial cancer risk.^10^ Lower circulating levels of adiponectin and higher levels of leptin have been associated with an increased risk for endometrial cancer.^11^, ^12^, ^13^ Additionally, a higher adiponectin‐leptin ratio (A:L ratio) has been associated with a reduced risk of endometrial cancer.^12^ Studies of the association between the pro‐inflammatory cytokine C‐reactive protein (CRP) with endometrial cancer incidence have been mixed,^9^, ^14^, ^15^, ^17^ and meta‐analyses of tumor necrosis factor alpha (TNF‐α) and interleukin 6 (IL‐6) have not demonstrated an association with endometrial cancer risk.^11^, ^13^

Although metabolic dysregulation and chronic inflammation have been linked to the development of endometrial cancer, few studies have examined their impact on endometrial cancer prognosis.^18^, ^19^, ^20^, ^21^ In endometrial cancer survivors, obesity has been associated with increased cancer‐specific and all‐cause mortality^22^ whereas, physical activity has been associated with improved disease‐free and overall survival.^23^ However, the biological mechanisms underlying these relationships have not been investigated. Moreover, cancer treatments may lead to endocrine dysregulation and promote insulin resistance and chronic inflammation, which may consequently lead to cancer recurrence or the development of chronic diseases as a late effect of treatment.^24^ Therefore, the objective of this report was to examine the associations of insulin resistance and inflammatory biomarkers with disease‐free survival and overall survival in a prospective follow‐up of women with incident endometrial cancer who previously participated in our case–control study.

## 2 METHODS

### 2.1 Design

The Alberta Endometrial Cancer Cohort Study was a prospective study of incident endometrial cancer cases from a previous population‐based case–control study conducted in Alberta, Canada. The methods for the case–control study have been described in detail elsewhere.^25^ Briefly, 549 women with histologically confirmed invasive endometrial cancer were identified through the Alberta Cancer Registry (ACR) from 2002 to 2006. Inclusion criteria for cases were: (1) Alberta resident, (2) <80 years of age, (3) English speaking, (4) no previous history of cancer (except for non‐melanoma skin cancer), and (5) willing and able to complete an in‐person interview and diet history questionnaire. Participants were excluded from the current analyses if they had an ineligible endometrial cancer subtype (*n* = 1), their cancer diagnosis had been misclassified (*n* = 1), their baseline interview was incomplete (*n* = 7), or they did not provide a blood sample (*n* = 20). The final sample in the present analysis consisted of 520 cases. Ethics approval for this study was obtained from the former Alberta Cancer Board, the Conjoint Health Research Ethics Board (University of Calgary), and the Health Research Ethics Board (University of Alberta). All participants provided written informed consent prior to study participation.

### 2.2 Data collection

Data collection methods have been described elsewhere.^25^ Briefly, detailed demographic (age, race, education, residential status, marital status), health (parity, menopausal status, hormone use, family history of uterine or colorectal cancer, co‐morbidities), and behavioral characteristics (lifetime smoking habits and physical activity) were collected via in‐person interviews. Lifetime alcohol consumption and caloric intake were assessed using the self‐administered Canadian Diet History Questionnaire‐I.^26^ Anthropometrics (height, weight, waist, and hip circumference) were obtained via direct measures during interviews completed after diagnosis (mean 22 ± 15 weeks).^25^ Fasting (minimum 8 h) blood samples were collected from participants at several sites across Alberta prior to surgery (*n* = 235) or 4–6 weeks after surgery (*n* = 285) when it was not possible to draw blood before surgery. Blood samples were processed into blood fractions (serum, plasma, red blood cells, and buffy coat) and frozen at −86°C within 24 h of collection. All blood samples were transported to the Tom Baker Cancer Centre in Calgary, Alberta where they were stored in a biorepository.

#### 2.2.1 Laboratory assays

Blood processing details have been reported in detail elsewhere.^8^, ^9^ Briefly, blood samples were analyzed in the laboratory of Dr. David Lau at the University of Calgary by a single technician who was blinded to case–control status. Plasma concentrations of glucose were measured by fluorimetric quantitative determination (Bioassay Systems). Serum concentrations of insulin were measured by RIA (Linco Research), adiponectin and leptin by ELISA (Alpo Diagnostics), and TNF‐α, IL‐6 and CRP by solid‐phase sandwich enzyme‐linked immunosorbent assays (Alpo Diagnostics). Assays were analyzed in batches of 72 samples (1 case: 2 controls) in the sequence of data collection. The mean intra‐ and inter‐batch coefficients of variation were 3.7% and 4.6% for glucose, 5.0% and 5.3% for insulin, 4.6% and 5.6% for adiponectin, 3.4% and 7.9% for leptin, 5.9% and 8.5% for TNF‐α, 6.4% and 6.7% for IL‐6, and 5.5% and 6.6% for CRP, respectively. The homeostasis model assessment for insulin resistance (HOMA‐IR), which uses fasting measures of insulin and glucose to estimate insulin resistance was calculated as: [fasting insulin (mIU/L) × fasting glucose (mg/dl)]/405.

#### 2.2.2 Chart abstractions and vital status

Clinical data including cancer histology, cancer stage, cancer grade, cancer treatments, and cancer recurrence or progression were abstracted from medical records through the ACR. Cancer stage was determined using the American Joint Committee on Cancer guidelines.^27^ Cancer grade was obtained from pathology reports where cancer grade was reported in accordance with the International Federation of Gynecology and Obstetrics guidelines as previously described.^28^ Vital status and cause of death were obtained from the ACR which obtains these data through record linkage with Vital Statistics Alberta and Statistics Canada. Participants were followed from the date of their endometrial cancer diagnosis until death or March 20, 2019, whichever occurred first. In the current analyses, disease‐free survival was defined as the time from diagnosis to the first recurrence, progression, or death from any cause. Overall survival was defined as the time from diagnosis to death from any cause.

### 2.3 Statistical analyses

Cox proportional hazard regression models were used to estimate multivariable‐adjusted hazard ratios (HR) and 95% confidence intervals (95% CI) for the associations between each biomarker (continuous and according to tertiles) and disease‐free survival and overall survival. Covariates included in the models based on biological plausibility were: age at diagnosis (years), cancer stage (I; II; III/IV), cancer grade (I/II; III; unknown), cancer treatment (hysterectomy only; hysterectomy/chemotherapy; hysterectomy/radiation therapy; hysterectomy/chemotherapy/radiation therapy and/or hormone therapy; treatment not received), type 2 diabetes (yes/no), hypertension (yes/no), and number of other co‐morbidities (0;1;2). Additional covariates included in the final models based on backwards elimination were waist circumference (cm), smoking (pack‐years), and residence (urban; rural). There was insufficient evidence that age^2^, education (high school or less; trade or non‐university diploma; university degree), marital status (married/common law; other), parity (null; 1–2; >2), menopausal status (pre/peri‐menopausal; post‐menopausal), hormone use (ever; never), family history of uterine or colorectal cancer (yes; no), total alcohol consumption (g ethanol/year), total caloric intake (kcal/day), or lifetime total physical activity (MET h/week/year) confounded associations between the biomarkers and survival outcomes. Missing values for covariates (<1%) were replaced with the mode for categorical variables and the mean for continuous variables. The proportional hazards assumption was evaluated by statistical and visual assessment of the Schoenfeld residuals. Results for analyses that violated the proportional hazards assumption are not presented. The associations between the biomarkers and survival outcomes were examined by timing of blood collection (pre‐ vs. post‐surgical) in an exploratory stratified analysis. A sensitivity analysis was conducted to assess how results changed when excluding women who self‐reported taking antihyperglycemic medication (i.e., Metformin hydrochloride, Gliclazide, Glyburide, Insulin Regular Human, Insulin Toronto, other) for type 2 diabetes. All analyses were performed with STATA 16 (StataCorp LLC.).

## 3 RESULTS

The full cohort has been described in detail elsewhere.^23^ Characteristics of the 520 women included in the current analyses are presented in Table 1. At baseline, the median age of participants was 59 years (interquartile range 53–65 years), 69% were married, 77% were post‐menopausal, and median body mass index (BMI) was 31.0 kg/m^2^ (interquartile range 26.4–37.0 kg/m^2^). Most participants were diagnosed with stage 1 (80%) and low grade (54%) endometrial cancer and had a hysterectomy as their primary treatment (98%). During the median follow‐up period of 14.3 years (range 0.4–16.5 years), there were 125 recurrences, progressions, and/or deaths with 106 overall deaths.

**TABLE 1:** Baseline descriptive characteristics of the Alberta Endometrial Cancer Cohort Study by vital status, 2002–2019 (*N* = 520)

Characteristics	All	Alive	Disease‐free survival events	Overall deaths
Median (IQR), *n* (%)	*N* = 520	*n* = 414	*n* = 125	*n* = 106
*Demographic profile*				
Age at diagnosis, years	59 (53–65)	58 (53–64)	64 (58–72)	66 (59–73)
Highest education				
High school diploma	111 (21)	95 (23)	20 (16)	16 (15)
Non‐university certificate	238 (46)	189 (46)	57 (46)	49 (46)
University degree	171 (21)	130 (31)	48 (38)	41 (39)
Married or common law	361 (69)	292 (71)	80 (64)	69 (65)
Urban residence	352 (68)	293 (71)	73 (58)	59 (56)
White	495 (95)	393 (95)	118 (94)	102 (96)
Parity				
0	108 (21)	89 (22)	26 (21)	19 (18)
1–2	235 (45)	197 (48)	44 (35)	38 (36)
>2	177 (34)	128 (31)	55 (44)	49 (46)
Menopausal status				
Pre‐ and peri‐menopausal	121 (23)	114 (28)	15 (12)	7 (7)
Post‐menopausal	399 (77)	300 (72)	110 (88)	99 (93)
Ever had hormone replacement therapy	236 (45)	192 (46)	50 (40)	44 (42)
*Medical profile*				
Histology				
Endometrioid	424 (82)	353 (85)	87 (70)	71 (67)
Non‐endometrioid	96 (18)	96 (15)	38 (30)	35 (29)
Overall AJCC Stage				
I	416 (80)	352 (85)	76 (61)	64 (60)
II	63 (12)	45 (11)	22 (18)	18 (17)
III/IV	41 (8)	17 (4)	27 (22)	24 (23)
FIGO grade				
<6%	278 (53)	242 (58)	45 (36)	36 (34)
6%–50%	119 (23)	97 (23)	26 (21)	22 (21)
>50%	70 (13)	39 (9)	33 (26)	31 (30)
Other	53 (10)	36 (9)	21 (17)	17 (16)
Primary treatment				
Hysterectomy	507 (98)	407 (98)	113 (90)	100 (94)
Chemotherapy	44 (8)	26 (6)	19 (15)	18 (17)
Radiation therapy	159 (31)	118 (29)	48 (38)	41 (39)
Hormone therapy	6 (1)	6 (1)	2 (2)	0 (0)
Not received	30 (6)	14 (3)	23 (18)	16 (15)
Family history of uterine or colorectal cancer	87 (17)	63 (15)	27 (22)	24 (23)
Ever had type 2 diabetes	61 (12)	42 (10)	21 (17)	19 (18)
Ever had hypertension	219 (42)	160 (39)	65 (52)	59 (56)
Number of other comorbidities^a^				
0	305 (59)	257 (62)	59 (47)	48 (45)
1	184 (35)	139 (34)	53 (42)	45 (42)
≥2	31 (6)	18 (4)	13 (10)	13 (12)
Weight, kg	81.2 (68.6–98.1)	81.6 (68.5–99.2)	80.8 (69.4–96.0)	80.2 (69.4–96.0)
Waist circumference, cm	95.5 (84.2–108.3)	95.0 (83.8–108.0)	96.0 (87.0–110.0)	96.1 (86.0–108.5)
BMI (kg/m^2^)	31.0 (26.4–37.0)	31.1 (26.3–37.1)	31.0 (27.4–36.0)	30.9 (27.2–35.7)
Glucose, mg/dl	113.9 (90.7–144.9)	114.0 (90.9–144.4)	112.8 (90.7–147.5)	112.5 (89.5–147.5)
Insulin, pmol/L	46.4 (29.1–75.5)	43.4 (28.3–74.7)	56.3 (32.9–83.7)	54.3 (32.6–83.7)
Adiponectin, μg/ml	11.5 (7.8–17.3)	11.6 (7.6–17.1)	10.9 (7.9–18.4)	11.1 (8.0–19.3)
Leptin, ng/ml	44.3 (23.5–72.9)	43.2 (23.4–69.5)	49.0 (25.4–82.2)	50.4 (24.8–84.6)
Adiponectin: leptin ratio	0.28 (0.13–0.61)	0.28 (0.13–0.63)	0.23 (0.12–0.54)	0.23 (0.12–0.56)
HOMA‐IR^b^	1.80 (1.12–3.19)	1.76 (1.09–3.13)	2.10 (1.37–3.42)	2.08 (1.29–3.72)
Tumor necrosis factor α, pg/ml	4.2 (3.2–5.4)	4.2 (3.1–5.3)	4.2 (3.4–5.7)	4.3 (3.5–5.8)
Interleukin‐6, pg/ml	2.4 (1.6–3.5)	2.3 (1.6–3.5)	2.5 (1.7–3.7)	2.5 (1.7–3.7)
C‐reactive protein, μg/ml	3.8 (2.3–6.2)	3.9 (2.2–6.3)	3.8 (2.6–5.5)	3.8 (2.6–5.5)
Triglycerides, mg/dl	118.4 (79.1–184.2)	116.3 (78.4–178.8)	128.4 (85.7–212.9)	132.4 (82.6–209.7)
High‐density lipoprotein, mg/dl	38.7 (31.4–45.6)	39.1 (32.4–45.5)	36.9 (27.1–45.1)	36.9 (26.1–45.7)
*Behavioral profile*				
Smoker (ever)	255 (49)	205 (49)	58 (46)	50 (47)
Smoking status				
Nonsmoker	265 (51)	209 (50)	67 (54)	56 (53)
Current smoker	69 (13)	49 (12)	21 (17)	20 (19)
Ex‐smoker	164 (32)	137 (33)	34 (27)	27 (25)
Occasional smoker	22 (4)	19 (5)	3 (2)	3 (3)
Smoking, pack‐years^c^	13.2 (2.1–30.2)	11.7 (1.5–28.2)	20.2 (6.3–38.7)	20.2 (6.3–39.2)
Alcohol consumption, g ethanol/year	300.3 (26.7–1045.8)	351.0 (43.1–1159.0)	138.6 (0–636.4)	127.9 (0–559.6)
Total caloric intake, kcal/day	1497.1 (1159.1–1892.8)	1503.9 (1190.4–1894.8)	1460.4 (1057.0–1884.5)	1468.3 (1057.0–1892.1)
Lifetime physical activity, MET h/week/year				
Total	116.3 (96.6–137.0)	116.7 (97.2–136.7)	114.0 (94.4–138.9)	114.0 (92.7–144.1)
Recreational	10.7 (6.9–16.8)	10.9 (7.3–16.8)	9.7 (6.3–17.0)	9.5 (6.2–17.0)

Abbreviations: AJCC, American Joint Committee on Cancer; BMI, body mass index; FIGO, International Federation of Gynecology and Obstetrics; HOMA‐IR, homeostatic model assessment for insulin resistance; IQR, interquartile range.

^a^ Other comorbidities include hyperlipidemia, myocardial infarction, angina pectoris, pulmonary embolism, thrombosis, and stroke.

^b^ Insulin in mIU/L and glucose in mg/dl.

^c^ For current smokers, ex‐smokers, and occasional smokers.

There were no significant associations between any of the biomarkers and disease‐free survival or overall survival in the multivariable‐adjusted models (Table 2). The models assessing associations between insulin and disease‐free survival did not satisfy the proportional hazards assumption and are therefore not reported. When examining associations by the timing of blood collection, compared with the lowest tertile of presurgical adiponectin (T1 ≤ 8.9 μg/ml), the highest tertile (T3 > 14.9 μg/ml) was associated with improved disease‐free survival (HR = 0.42, 95% CI = 0.20–0.85; p=0.016) and overall survival (HR = 0.41, 95% CI = 0.18–0.92; p=0.031) whereas, no statistically significant associations were noted for postsurgical measures of adiponectin (Table 3). No other statistically significant associations were observed between any of the biomarkers and disease‐free and overall survival after stratifying the results by timing of the blood collection. When women who self‐reported taking antihyperglycemic medication for type 2 diabetes were excluded from the analyses (*n* = 43), increasing levels of leptin were associated with worse disease‐free survival (HR_per 5 μg/ml_ = 1.04, 95% CI = 1.00–1.07) and overall survival (HR_per 5 μg/ml_ = 1.05, 95% CI = 1.01–1.09). No other statistically significant associations were observed.

**TABLE 2:** Associations of insulin resistance and inflammatory biomarkers with disease‐free survival and overall survival in the Alberta Endometrial Cancer Cohort Study, 2002–2019 (*N* = 520)

	Disease‐free survival		Overall survival	
	Events/Cases	Multivariable HR (95% CI)	Events/Cases	Multivariable HR (95% CI)
Glucose mg/dl				
≤97.9	—	—	36/174	
>97.9 to ≤132.9	—	—	31/173	0.60 (0.36–1.00)
>132.9	—	—	39/173	0.71 (0.43–1.18)
Per 5 mg/dl	—	—	106/520	0.98 (0.95–1.00)
Insulin, pmol/L				
≤34.9	35/174		31/174	
>34.9 to ≤62.1	49/173	1.03 (0.64–1.67)	35/173	0.80 (0.48–1.35)
>62.1	41/173	0.95 (0.56–1.61)	40/173	0.81 (0.45–1.45)
Per 5 pmol/L	125/520	1.00 (0.98–1.02)	106/520	1.00 (0.97–1.02)
HOMA‐IR^a^				
≤1.3	30/174		27/174	
>1.3 to ≤2.7	46/173	1.12 (0.73–1.93)	40/173	1.02 (0.61–1.72)
>2.7	49/173	0.95 (0.56–1.62)	39/173	0.81 (0.46–1.44)
Per 0.1 unit of change	125/520	1.00 (0.99–1.00)	106/520	1.00 (0.99–1.00)
Adiponectin, μg/ml				
≤8.9	44/174		36/174	
>8.9 to ≤14.9	41/175	0.86 (0.54–1.36)	32/175	0.89 (0.54–1.49)
>14.9	40/171	0.79 (0.48–1.31)	38/171	0.94 (0.54–1.61)
Per 5 μg/ml	125/520	1.01 (0.89–1.15)	106/520	1.07 (0.94–1.21)
Leptin, ng/ml				
≤27.8	37/174		31/174	
>27.8 to ≤60.3	38/173	0.85 (0.51–1.41)	31/173	0.93 (0.53–1.63)
>60.3	50/173	1.05 (0.60–1.86)	44/173	1.27 (0.69–2.34)
Per 5 μg/ml	125/520	1.01 (0.98–1.04)	106/520	1.02 (0.99–1.05)
Adiponectin: leptin ratio				
≤0.17	47/174		39/174	
>0.17 to ≤0.43	41/173	0.96 (0.60–1.54)	34/173	0.88 (0.52–1.49)
>0.43	37/173	0.90 (0.50–1.62)	33/173	0.89 (0.46–1.70)
Per 0.1 unit of change	125/520	0.99 (0.98–1.01)	106/520	1.00 (0.98–1.01)
Tumor necrosis factor α, pg/ml				
≤3.6	38/174		30/174	
>3.6 to ≤4.9	41/174	0.83 (0.53–1.32)	34/174	0.85 (0.51–1.42)
>4.9	46/172	0.77 (0.46–1.28)	42/172	1.07 (0.62–1.83)
Per 5 pg/mL	125/520	0.81 (0.46–1.41)	106/520	1.18 (0.68–2.04)
Interleukin‐6, pg/ml				
≤1.9	41/174		35/174	
>1.9 to ≤3.2	38/176	0.64 (0.40–1.05)	32/176	0.98 (0.41–1.14)
>3.2	46/170	0.79 (0.48–1.30)	39/170	0.90 (0.54–1.51)
Per 5 pg/mL	125/520	0.87 (0.40–1.90)	106/520	0.99 (0.45–2.17)
C‐reactive protein, ug/ml				
≤2.9	37/174		34/174	
>2.9 to ≤5.1	48/174	0.84 (0.51–1.38)	39/173	0.77 (0.44–1.32)
>5.1	40/173	0.66 (0.37–1.16)	33/173	0.62 (0.34–1.14)
Per 5 μg/ml	125/520	0.96 (0.79–1.16)	106/520	0.95 (0.77–1.17)

*Note*: Adjusted for age, cancer stage (I; II; III/IV), cancer grade (I/II; III; unknown), cancer treatment (hysterectomy only; hysterectomy/chemotherapy; hysterectomy/radiation therapy; hysterectomy/chemotherapy/radiation therapy and/or hormone therapy; treatment not received), type 2 diabetes (yes; no), hypertension (yes; no), number of other comorbidities (0; 1; ≥2), waist circumference (cm), smoking pack‐years, and residence (urban; rural).

Abbreviation: HOMA‐IR, homeostatic model assessment for insulin resistance.

^a^ Insulin in mIU/L and glucose in mg/dl.

**TABLE 3:** Associations of adiponectin with disease‐free survival and overall survival, stratified by timing of blood assessment in the Alberta Endometrial Cancer Cohort Study, 2002–2019 (*N* = 520)

	Disease‐free survival				Overall survival			
	Presurgical		Postsurgical		Presurgical		Postsurgical	
	Events/cases	Multivariable HR (95% CI)	Events/cases	Multivariable HR (95% CI)	Events/cases	Multivariable HR (95% CI)	Events/cases	Multivariable HR (95% CI)
Adiponectin, μg/ml								
≤8.9	25/72		19/102		20/72		16/86	
>8.9 to ≤14.9	28/87	0.71 (0.39–1.27)	13/88	0.97 (0.47–2.00)	22/87	0.73 (0.38–1.41)	10/78	0.97 (0.43–2.17)
>14.9	17/76	0.42 (0.20–0.85)^*^	23/95	1.37 (0.71–2.64)	15/61	0.41 (0.18–0.92)^**^	23/72	1.74 (0.88–3.44)
Per 5 μg/ml	70/235	0.92 (0.76–1.10)	55/285	1.10 (0.94–1.29)	57/235	0.94 (0.77–1.15)	49/285	1.16 (0.96–1.34)

*Note*: Adjusted for age, cancer stage (I; II; III/IV), cancer grade (I/II; III; unknown), cancer treatment (hysterectomy only; hysterectomy/chemotherapy; hysterectomy/radiation therapy; hysterectomy/chemotherapy/radiation therapy and/or hormone therapy; treatment not received), type 2 diabetes (yes; no), hypertension (yes; no), number of other comorbidities (0; 1; ≥2), waist circumference (cm), smoking pack‐years, and residence (urban; rural).

^*^ p=0.016

^**^ p=0.031

## 4 DISCUSSION

Overall, there was no evidence of an association between biomarkers of insulin resistance and inflammation with mortality outcomes in a cohort of endometrial cancer survivors. After stratifying by the timing of blood collection, the highest tertile of adiponectin, compared to the lowest tertile, was associated with improved disease‐free survival and overall survival in blood samples collected presurgery, but not postsurgery. When women who self‐reported taking antihyperglycemic medication for type 2 diabetes were excluded from the analyses, increasing levels of leptin were associated with reduced disease‐free survival and overall survival.

To date, only two studies have examined the relationship between insulin resistance and endometrial cancer survival and did not observe associations between presurgical serum concentrations of insulin and recurrence^18^ or overall survival.^19^ In cohorts of female breast cancer survivors with assessments of biomarkers up to 1 year postsurgery, higher levels of fasting insulin, compared to lower levels, have been associated with increased recurrences,^29^, ^30^, ^31^ cancer‐specific deaths,^32^ and all‐cause mortality.^30^ Conversely, high versus low levels of fasting glucose have not been associated with recurrence in postmenopausal women previously operate on for breast cancer (HR = 2.42; 95% CI = 0.90–6.53)^29^ or with progression‐free survival in female breast cancer survivors assessed prior to receiving any treatment (HR = 0.82; 95% CI = 0.44–1.51).^31^ Increasing HOMA‐IR indices have been associated with increased breast cancer progression^31^ and reduced breast cancer‐specific and overall survival.^33^ Additionally, in a retrospective chart review of women diagnosed with early‐stage cervical cancer, impaired fasting glucose (≥100 mg/dl) measured at the time of diagnosis and prior to surgery was associated with a higher risk of recurrence (HR = 4.30; 95% CI = 1.23–15.03).^34^

Although there is evidence to support the role of insulin resistance in the promotion and progression of cancer,^4^ we did not observe any associations between insulin, glucose, and the HOMA‐IR index with disease‐free survival or overall survival. Endometrial cancer has a relatively high 5‐year survival rate (83% in Canada)^3^ thus, endometrial cancer survivors are more likely to die from other causes which may partially explain why insulin resistance was not associated with survival outcomes in our study. Furthermore, comorbid conditions such as obesity, diabetes, hypertension, and dyslipidemia, may be stronger predictors of mortality outcomes in this population. This is supported by previous reports from the Alberta Endometrial Cancer Cohort Study in which measures of obesity were associated with reduced survival.^35^, ^36^ Moreover, metabolic syndrome was associated with worse disease‐free and overall survival in endometrial cancer survivors.^35^

Only one study to date has examined the relationship between adipokines (leptin and adiponectin) and endometrial cancer survival and did not observe an association between adiponectin levels and overall survival in women with type 1 endometrial cancer.^19^ In breast cancer cohorts, higher levels of adiponectin measured prior to surgery^37^, ^38^, ^39^ and approximately 24 months post‐diagnosis^33^ have been associated with better prognosis; however, no associations have been found for leptin.^30^, ^37^, ^38^, ^40^ In the current study, higher levels of presurgical adiponectin were associated with improved survival outcomes; however, no associations were found for adiponectin measured postsurgery. Given the paucity of evidence, there is no clear explanation for this discrepancy. However, it may be hypothesized that adiponectin measured soon after hysterectomy is not a stable prognostic indicator given the multitude of physiological changes that occur with this major surgery (i.e., stress response to trauma, hormonal imbalances from the removal of ovaries, and weight changes), all of which may influence circulating levels of adiponectin. It is also unclear why only increasing levels of leptin were associated with reduced disease‐free survival and overall survival after excluding women taking antihyperglycemic medication for type 2 diabetes. These findings should be interpreted with caution due to the exploratory nature of these analyses.

A limited number of studies have examined the relationship between inflammatory biomarkers and endometrial cancer survivors; nevertheless, findings have consistently demonstrated an inverse association between circulating levels of CRP and endometrial cancer survival.^19^, ^20^, ^21^ No studies have examined the influence of circulating levels of IL‐6 or TNF‐α on prognostic outcomes for endometrial cancer survivors. In breast cancer cohorts, CRP has been associated with reduced disease‐free and overall survival^41^, ^42^, ^43^, ^44^ and IL‐6 has been associated with reduced overall survival.^45^, ^46^ Only two studies have examined the influence of TNF‐α on outcomes in cancer survivors and have found an association between higher levels, compared to lower levels, with worse progression‐free and overall survival in breast cancer survivors^46^ and increased recurrence in esophageal cancer survivors.^47^ Our null findings are contrary to existing research and challenging to interpret. Previous research examining the relationship between inflammatory biomarkers and cancer survival have not considered important variables including obesity and comorbidities in their analyses which may partially explain their positive associations. Similar to metabolic dysregulation, inflammation may be a predictor of chronic diseases which, in turn, may be more strongly associated with mortality outcomes.

Strengths of the current study include the large population‐based cohort of incident endometrial cancer cases, detailed assessments of covariates, direct measures of anthropometric outcomes, and long‐term follow‐up which is required given the relatively high 5‐year survival rate in this population.^3^ This study has notable limitations including the relatively small cohort and number of events, possible measurement error, and the collection of blood at only one timepoint, all of which may have hindered our ability to detect associations. Moreover, we conducted multiple analyses without adjustment which increases the likelihood that our statistically significant findings for our exploratory and sensitivity analyses were due to chance. Finally, given that our sample of endometrial cancer survivors was relatively homogenous for several characteristics including age, race, ethnicity, and obesity status, our findings may not be generalizable.

Although metabolic dysregulation and inflammation are associated with endometrial cancer risk, their impact on endometrial cancer survival has not been established. In the current study, there was no evidence of a direct association between biomarkers of insulin resistance and inflammation with survival outcomes in endometrial cancer survivors. There was evidence that timing of the blood collection relative to surgery and antihyperglycemic medication for type 2 diabetes may influence the associations between adiponectin and leptin, respectively, and endometrial cancer survival outcomes. Future cohort studies with repeated assessments of blood biomarkers are needed to examine these associations more reliably given the potential influence of the tumor and cancer treatments on these measures.

## CONFLICT OF INTEREST

The authors have no conflict of interest to disclose.

## AUTHOR CONTRIBUTIONS

All authors were involved in the conceptualization of this project; Christine M. Friedenreich, Linda S. Cook, and Kerry S. Courneya designed the project methodology, conducted this research investigation, and acquired the financial support for the project. Linda S. Cook and Kerry S. Courneya were responsible for project administration and Christine M. Friedenreich managed activities to maintain research data. Andria R. Morielli conducted the formal analysis and the original draft of the manuscript. All authors reviewed and edited the manuscript.

## ETHICS APPROVAL AND CONSENT TO PARTICIPATE

This study was approved by the former Alberta Cancer Board, the Conjoint Health Research Ethics Board (University of Calgary), and the Health Research Ethics Board (University of Alberta), and written informed consent was obtained from all participants. This study was conducted in accordance with the Declaration of Helsinki.

## Data Availability

The data underlying this article will be shared on reasonable request to the corresponding author.
